# Efficient Removal of Metals from Synthetic and Real Galvanic Zinc–Containing Effluents by Brewer’s Yeast *Saccharomyces cerevisiae*

**DOI:** 10.3390/ma13163624

**Published:** 2020-08-16

**Authors:** Inga Zinicovscaia, Nikita Yushin, Daler Abdusamadzoda, Dmitrii Grozdov, Margarita Shvetsova

**Affiliations:** 1 Department of Nuclear Physics, Joint Institute for Nuclear Research, Joliot-Curie Street 6, Dubna 1419890, Russia; ynik_62@mail.ru (N.Y.); martinez-91@mail.ru (D.A.); dsgrozdov@rambler.ru (D.G.); mks@nf.jinr.ru (M.S.); 2 Department of Nuclear Physics, Horia Hulubei National Institute for R&D in Physics and Nuclear Engineering, 30 Reactorului, MG-6 Bucharest-Magurele, Romania; 3 Laboratory of Quantum Chemistry, Catalysis and Physical Methods, The Institute of Chemistry, Academiei Street 3, 2028 Chisinau, Moldova

**Keywords:** *Saccharomyces cerevisiae*, metal ions, industrial effluent, neutron activation analysis, ICP-MS, FTIR

## Abstract

The performance of the brewer’s yeast *Saccharomyces cerevisiae* to remove metal ions from four batch systems, namely Zn(II), Zn(II)-Sr(II)-Cu(II), Zn(II)-Ni(II)-Cu(II), and Zn(II)-Sr(II)-Cu(II)-Ba(II), and one real effluent was evaluated. Yeast biosorption capacity under different pH, temperature, initial zinc concentration, and contact time was investigated. The optimal pH for removal of metal ions present in the analyzed solution (Zn, Cu, Ni, Sr, and Ba) varied from 3.0 to 6.0. The biosorption process for zinc ions in all systems obeys Langmuir adsorption isotherm, and, in some cases, the Freundlich model was applicable as well. The kinetics of metal ions biosorption was described by pseudo-first-order, pseudo-second-order, and Elovich models. Thermodynamic calculations showed that metal biosorption was a spontaneous process. The two-stage sequential scheme of zinc ions removal from real effluent by the addition of different dosages of new sorbent allowed us to achieve a high efficiency of Zn(II) ions removal from the effluent. FTIR revealed that OH, C=C, C=O, C–H, C–N, and NH groups were the main biosorption sites for metal ions.

## 1. Introduction

Environment pollution with heavy metals is one of the most serious problems, since metals, even in traces, can be toxic and detrimental for living organisms [1]. Zinc is an essential element involved in different metabolic processes and an important component of more than 200 enzymes [2]. At the same time, zinc compounds are widely used in different industrial processes, which often result in the release of zinc ions into the environment, at a concentration of physiological and ecological concern [3]. Wastewater released by different industrial processes (electroplating and surface finishing) and mining contain high concentrations of zinc among other metal ions [4].

Conventional technologies, which include flocculation, precipitation, ion exchange, membrane separation, and solvent extraction, are applied for the treatment of wastewaters containing metal ions. However, these processes are usually expensive and show low efficiency of metal removal from slightly contaminated effluents which contain relatively low metal concentrations [1,3,4]. Biosorption can be considered to be a cost-effective technique for wastewater treatment, using either live or dead microorganisms [1].

Yeast cells of *Saccharomyces cerevisiae* (*S. cerevisiae*) are considered good candidates for wastewater treatment. Application of *S. cerevisiae* as a biosorbent is attributable to yeast safety, availability in large quantities at a very low cost, and high metal uptake capacity [1,4]. *S. cerevisiae* is extensively used in beverage and food production and is obtained in large quantities, as a by-product of the fermentation industry [5]. It was already shown that *S. cerevisiae* can remove toxic metals and radionuclides from aqueous solutions [1,3,4,5,6,7]. In the Ozer and Ozer [7] study, inactive *S. cerevisiae* biomass showed the highest sorption capacity for Pb(II) ions, followed by Ni(II) and Cr(VI). *S. cerevisiae* crosslinked with formaldehyde was used for Cu^2+^, Zn^2+^, and Cd^2+^ removal from solution. The metal uptake capacities of the biomass for Cu^2+^, Zn^2+^, and Cd^2+^ were found to be 8.0, 7.1, and 14.0 mg/g, respectively [8]. Sorption properties of baker’s yeast cells *S. cerevisiae* toward Cd^2+^, Cr^3+^, CrO_4_^2−^, Cu^2+^, Pb^2+^, and Zn^2+^ were evaluated [9]. It should be mentioned that metal removal was mainly investigated from a single-metal solution, whereas wastewater can be characterized as complex multi-metal systems in which metal removal efficiency will be different from single-metal solution due to the competition of metal ions for the binding sites on the biosorbent surface.

The present work is focused on the application of yeast *S. cerevisiae* for metal removal from four synthetic effluents with different chemical compositions and one real zinc-containing industrial effluent. For synthetic effluents, the effect of contact time, initial zinc concentration, temperature, and pH on the sorption process was investigated. The kinetic and equilibrium models were applied to describe experimentally obtained values. Thermodynamics parameters were calculated. For real effluent, the effect of pH and sorbent dosage on metal removal was investigated.

## 2. Materials and Methods

### 2.1. Chemicals

Chemical used in the present study—Ni(NO₃)_2_·6H₂O, Cu(NO_3_)_2_·2.5H_2_O, Zn(NO_3_)_2_·6H_2_O, Sr(NO_3_)_2_, and Ba(NO_3_)_2_—were purchased from Sigma-Aldrich (Moscow, Russia) and were of analytical grade.

### 2.2. Synthetic Effluents

Four synthetic effluents, namely Zn(II), Zn(II)-Sr(II)-Cu(II), Zn(II)- Ni(II)-Cu(II), and Zn(II)-Sr(II)-Cu(II)-Ba(II), were prepared from metal salt stock solutions of Ni(NO_3_)_2_·6H₂O, Cu(NO_3_)_2_·2.5H_2_O, Zn(NO_3_)_2_·6H_2_O, Sr(NO_3_)_2_, and Ba(NO_3_)_2_. Synthetic effluents were modeled based on the data obtained for real galvanic described in the [10] Zinicovscaia et al. (2019) study. The metal ions’ concentrations in synthetic effluents are presented in Table 1.

### 2.3. Industrial Effluent Characterization

The real effluent, containing zinc and other metal ions in different concentrations, was obtained from the electroplating company (Dubna, Russia). The chemical composition of the effluent and the initial pH are given in Table 2.

### 2.4. Preparation of Biosorbent

The yeast *Saccharomyces cerevisiae* used as sorbent was obtained from the company Efes Vitanta Moldova Brewery (Chisinau, Moldova). The procedure of sorbent preparation for analysis can be found in Reference [10].

### 2.5. Biosorption Experiments with Synthetic Effluents

In experiments with synthetic effluents, the effect of time (5–120 min), zinc concentration (10–100 mg/L), pH (2.0–6.0), and temperature (20–50 °C) on yeast sorption capacity was investigated. In total, 50 mL of synthetic effluents was added in 100 mL flasks containing 10 g/L of dried biomass. Samples were placed on the shaker and continuously agitated for 60 min. At the end of the experiment, biomass was separated from the solution by filtration, dried at 105 °C, weighted at analytical balance, and packed in aluminum foil cups for irradiation a the IBR-2 reactor.

### 2.6. Biosorption Experiments with Real Effluent

In the experiment with real effluent, the effect of pH and sorbent dosage on metal removal efficiency was studied. To assess the effect of pH on biomass sorption capacity, 10 g/L g of dry biomass was added to 50 mL of effluent in a 100 mL flask and continuously stirred for 60 min. The pH of the effluent varied from 2.0 to 6.0. The pH of the experimental solutions was adjusted to the required pH, using HNO_3_ or NaOH solutions. 

The experiment on the effect of the dosage of sorbent on metal removal was performed in two stages. On the first stage, sorbent in the dosages ranging from 20 to 40 g/L was added to 100 mL of effluent at initial effluent pH (6.0). The suspension was shaken at 200 rpm for 60 min, and then the sorbent was removed by filtration. The supernatant obtained for each sorbent dosage was divided into three parts. One part was used for ICP-MS analysis, while two others were used for the second stage of the experiment. On the second stage, 1.0 or 10 g/L of new biomass was added to the effluent, obtained after the first stage, and shacked for 60 min. Then, biomass was again separated from the supernatant by filtration. Metal concentration in obtained solutions was determined by using ICP-MS.

Experiments were performed in three repetitions, and the average values were used for discussion.

The metal uptake, *q*, was calculated by using Equation (1):

(1) $$q=\frac{V\left(C_{i}-C_{f}\right)}{m}$$

The sorption removal efficiency, *E*, (%) was calculated from Equation (2):

(2) $$E=\frac{C_{i}-C_{f}}{C_{i}}*100$$

where *q* is the amount of metal ions adsorbed on the biosorbent, mg/g; *V* is the volume of solution, ml; *C_i_* is the initial concentration of metal in mg/L; *C_f_* is the final metal concentration in the solution, mg/L; and *m* is the mass of sorbent, g.

### 2.7. Methods

To assess the effectiveness of zinc, nickel, strontium, and barium sorption from synthetic effluents neutron activation analysis at the pulsed fast reactor IBR-2 (Frank Laboratory of Neutron Physics, Joint Institute for Nuclear Research, Dubna, Russia) was used, while copper concentration in solution was determined by means of atomic absorption spectrometry. The procedure of samples irradiation and measurement is described in detail in Reference [10].

Metal concentration in the real effluent, before and after biosorption experiments, was determined by the mean of Element 2™ High-Resolution ICP-MS systems (The Thermo Scientific, Bremen, Germany). The operational parameters and settings are presented in Kuznetsova et al. [11].

Fourier-transform infrared spectroscopy (FTIR) was used to confirm the participation of functional groups in metal ions binding. The spectra were recorded by using a Nicolet 6700 spectrometer (Thermo Scientific, Madison, WI, USA).

## 3. Results

### 3.1. Metal Removal from Synthetic Effluents

#### 3.1.1. Effect of pH on Metal Ions’ Removal

**Figure 1:**
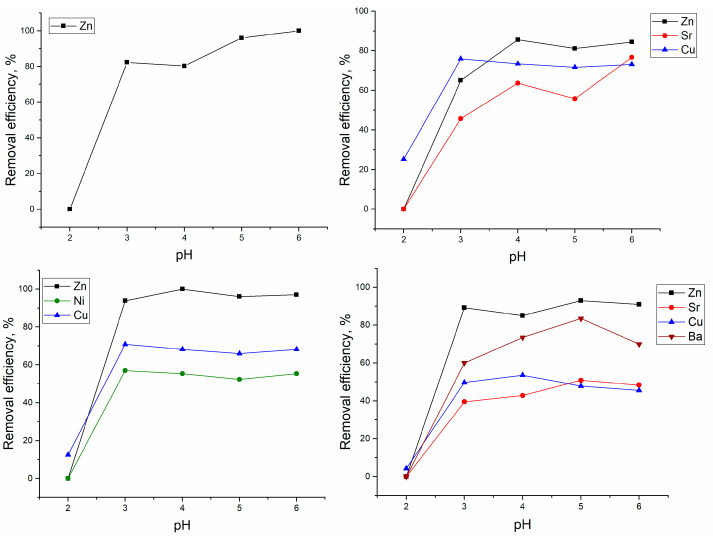
Effect of pH on yeast removal efficiency.

The sorption of Zn(II) ions in the Zn(II)-system increased with the pH increase, and it was completely removed from the solution at pH 6.0 (Figure 1).

In Zn(II)-Cu(II)-Sr(II) system, the optimal pH for Zn(II) removal lay in the range 3.0–6.0, and in comparison with a system containing only Zn(II) ions, its removal was reduced by 15% and constituted 84–86%. The maximum removal of Sr(II) in the same system was attained at pH 6.0 (77%), and of Cu(II) at pH 3.0 (76%). In the Zn(II)-Ni(II)-Cu(II) system, Zn(II) was completely removed from the solution at pH 4.0 and biomass maintained high Zn(II) removal efficiency at pH 5.0–6.0. The optimal pH for Ni(II) and Cu(II) removal was 3.0, and their removal was not affected by the pH increase. In the Zn(II)-Sr(II)-Cu(II)-Ba(II) system, the pH 5.0 was optimal for Zn(II), Sr(II), and Ba(II) ions’ removal, while Cu(II) ions were efficiently removed at pH 4.0.

#### 3.1.2. Effect of Time on Metal Removal and Kinetic Studies

**Figure 2:**
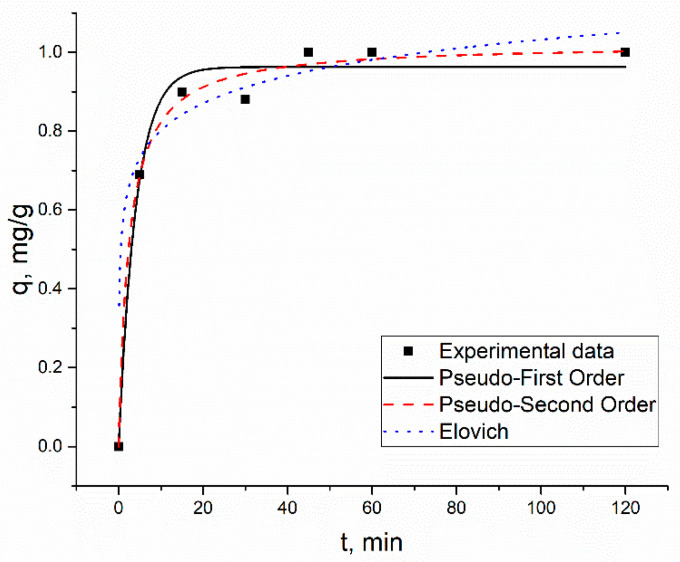
Pseudo-first-order (PFO), pseudo-second-order model (PSO), and Elovich model (EM) kinetics of the metal adsorption, using *S. cerevisiae*, in the Zn(II) system.

The biosorption of metal ions by *S. cerevisiae* was a time-dependent process. After 45 min of contact, Zn(II) ions were completely removed in the Zn(II) system (Figure 2).

Complete Zn(II) removal was also achieved in the Zn(II)-Cu(II)-Sr(II) system after 30 min of contact, and for the other two metal ions present in the solution, Sr(II) and Cu(II), maximum removal was attained in 45 min (Supplementary Materials Figure S1). In the case of Sr(II), after 30 min, the equilibrium was established, while the sorption of Cu(II) ions was reduced due to the competition with Zn(II) ions for the same functional groups. In the Zn(II)-Ni(II)-Cu(II) system, 1.2 mg/g (90%) of Zn(II) ions were removed in 30 min, and then the equilibrium was achieved. Cu(II) and Ni(II) were maximally taken from the solution in 30 min, and then the removal of both metal ions decreased (Supplementary Materials Figure S2). In the Zn(II)-Sr(II)-Cu(II)-Ba(II) system, 1.09 mg/g (89%) of the Zn(II) ions was removed in 30 min, and increasing the contact time to 2 h resulted in an increase of its removal by 5% and constituted 1.14 mg/g (94%). The maximum amount of Cu(II) ions, 0.12 mg/g (59%), was removed after 15 min of contact, and then it did not change after 2 h. The maximum amount of Sr(II) and Ba(II) ions was taken up from the solution after one hour of contract, 0.14 mg/g (70%) and 0.099 mg/g (100%), respectively (Supplementary Materials Figure S3).

The adsorption kinetic data were described by using three models: pseudo-first-order model (PFO), pseudo-second-order model (PSO), and the Elovich model (EM) The models are expressed by the following Equations (3)–(5): 

The pseudo-first-order model is reflected Equation (3):

(3) $$q\;=\;q_{e}(1-e^{-k_{1}t})$$

where *q_e_* and *q_t_* are the amounts of metal (mg/g) adsorbed at equilibrium and at *t* (min), time, respectively, and *k*_1_ (1/min) is the rate constant of pseudo-first-order. 

The pseudo-second-order model is reflected in Equation (4):

(4) $$q=\frac{q_{e}^{2}k_{2}t}{1+q_{e}k_{2}t}$$

where *k*_2_ (g/mg·min) is the rate constant of second order.

The Elovich model is reflected in Equation (5):

(5) $$q_{t\;}=\;\frac{1}{\beta }ln\left(1+\alpha \beta t\right)$$

where *α* (g/mg∙min) and *β* (g/mg) are the Elovich equation constants.

In addition to the coefficient of determination, the applicability of kinetic models was assessed by using the sum of error squares (SSE, %), given by Equation (6):

(6) $$\mathrm{SSE}=\frac{\sqrt{\sum \;{\left(q_{e,\;cal}-q_{e,\;exp}\right)}^{2}}}{N}$$

where *N* is the number of data points.

The modeling results of the applied models, as well as experimental data, are given in Supplementary Materials Figures S1–S3. Adsorption capacities values (experimental and calculated), as well as coefficients of determination, are listed in Table 3.

The experimentally obtained and calculated *q_e_* values for pseudo-first-order and pseudo-second-order models were in very good agreement. In the dependence of the analyzed system, different models were applicable to describe experimentally obtained data obtained for Zn(II) ions. In the Zn(II) and Zn(II)-Ni(II)-Cu(II) systems, Zn(II) ions’ adsorption followed the pseudo-second-order model. In the Zn(II)-Cu(II)-Sr(II) system pseudo-first-order and pseudo-second-order models were applicable to describe experimentally obtained data, while in the Zn(II)-Cu(II)-Sr(II)-Ba(II) system, it obeys the Elovich model. The Elovich model also better described data obtained for Cu(II) in the same system, in comparison with other applied models. For other elements present in analyzed systems, except for Cu(II) in Zn(II)-Cu(II)-Sr(II), the coefficients of determination obtained for pseudo-first-order and pseudo-second-order models were higher than 0.94, indicating the applicability of both models for the description of experimentally obtained data. 

#### 3.1.3. Effect of Zinc Concentration on Metal Ions Removal and Equilibrium Study

Equilibrium sorption studies were performed with different concentrations of Zn(II) ions from 10 to 100 mg/L. The increases in initial Zn(II) ions concentration increased the uptake of Zn(II) ions per unit weight of biosorbent (mg/g) and resulted in the decrease of yeast removal capacity. The maximum Zn(II) ions sorption in all analyzed systems was almost on the same level and lay in the range 5.34–6.1 mg/g. The removal of other metal ions present in the analyzed system was not dependent on Zn(II) concentration in solution and remained constant with its increase.

Langmuir, Freundlich, and Temkin isotherm models were used for the equilibrium modeling. The Langmuir model suggests monolayer adsorption and is expressed by Equation (7):

(7) $$q_{e}=\frac{q_{m\;}bC_{e}}{1+bC_{e}}$$

where *C_e_* is metal ions concentration at equilibrium (mg/L), *q_e_* is amount of metal adsorbed at equilibrium (mg/g), *q_m_* is maximum adsorption capacity of the sorbent (mg/g), and *b* is the Langmuir adsorption constant (L/mg) [10].

The mathematical expression of the Freundlich isotherm model is presented by Equation (8):

(8) $$q_{e}=K_{F}Ce^{\frac{1}{n}}$$

where *q_e_* is the amount of metal adsorbed at equilibrium (mg/g), *C_e_* is the concentration of metal ions in aqueous solution at equilibrium (mg/L), and *K_F_* and *n* are Freundlich constants that include factors that affect adsorption capacity and adsorption intensity, respectively [10].

The Temkin isotherm model is given below, in Equation (9):

(9) $$q_{e}=\frac{RT}{b_{T}}ln\left(a_{T}C_{e}\right)$$

where 1/*b_T_* indicates the sorption potential of the sorbent, *a_T_* is Temkin constant, *R* is the universal gas constant (8.314 J K^−1^ mol^−1^), and *T* is the temperature (K) [10].

**Figure 3:**
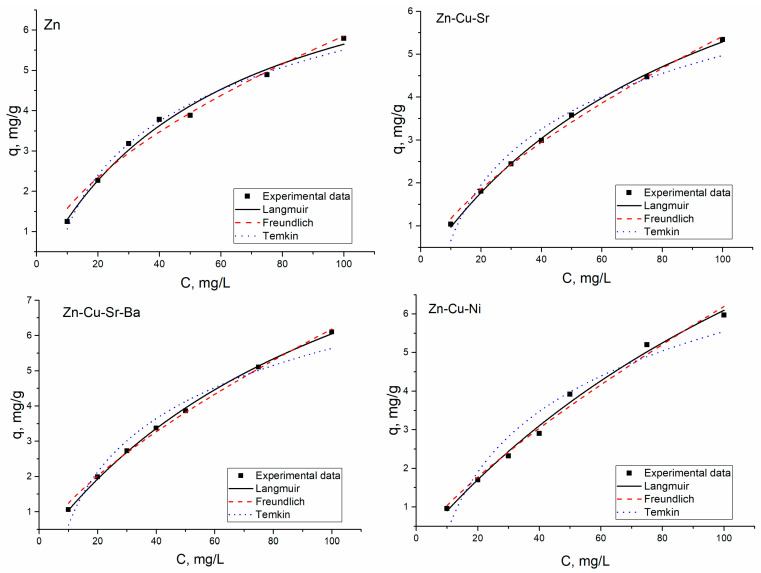
The adsorption isotherms for Zn(II) ions’ removal on *S. cerevisiae.*

The Langmuir, Freundlich, and Temkin constants were calculated by nonlinear regression (Figure 3).

The values of the parameters calculated from used models, as well as the coefficient of determination, are shown in Table 4.

High values of the coefficient of determination (*R*^2^ = 0.99) indicated that the adsorption of Zn(II) ions in all analyzed systems obey the Langmuir isotherm model. Zn(II) adsorption in Zn(II)-Cu(II)-Sr(II) and Zn(II)-Cu(II)-Sr(II)-Ba(II) systems also follow the Freundlich isotherm model. The Freundlich constant 1/n value less than 1.0 confirms the favorable adsorption [12]. The comparative analysis of *q_m_* values showed that yeast biomass accumulated more Zn(II) ions from Zn(II)-Ni(II)-Cu(II) system, in comparison with other analyzed systems. The coefficients of determination for the Temkin model were lower in comparison with those of the Langmuir and Freundlich models.

#### 3.1.4. Effect of Temperature on Metal Removal and Thermodynamic Studies

The uptake of metal ions was investigated at the temperature range of 20–50 °C. The yeast adsorption capacity for Zn(II) in Zn(II)-Ni(II)-Cu(II) and Zn(II)-Cu(II)-Sr(II)-Ba(II) systems and Cu(II) in all analyzed system was not dependent on the temperature grow. In Zn(II) and Zn(II)-Cu(II)-Sr(II) systems, an increase of temperature from 20 to 30 °C resulted in the increase of biomass sorption capacity by 5–18%, and then it did not change significantly. Sr(II) uptake by yeast occurred differently in the analyzed system; thus, in Zn(II)-Cu(II)-Sr(II), the temperature rise almost did not affect its removal, while in the Zn(II)-Cu(II)-Sr(II)-Ba(II) system, it decreased by 12%. Ni(II) and Ba(II) ions’ removal declined with the increase of temperature by 7% for Ni(II) and 20% for Ba(II).

The Gibbs free energy change (Δ*G*°), enthalpy change (Δ*H*°), and entropy change (Δ*S*°) values were calculated according to Equations (10)–(12), as presented below:

(10) $$\ln K_{d}\;=\;\frac{\Delta S^{0}}{R}-\frac{\Delta H^{0}}{RT}$$

(11) $$\Delta G^{0}\;=\;\Delta H^{0}-T\Delta S^{0}$$

where *K_d_* is the distribution coefficient, and it is calculated according to Equation (12).

(12) $$K_{d}=\frac{\left(C_{0}-C_{e}\right)V}{mC_{e}}$$

where *C*_0_ is the initial concentration of metal, (mg/L); *C_e_* is metal concentration in aqueous solution at equilibrium, (mg/L); *V* is the volume of aqueous solution (L); *m* is sorbent mass (g); *R* is the universal gas constant (8.314 J K^−1^ mol^−1^); and *T* is the temperature (K).

**Figure 4:**
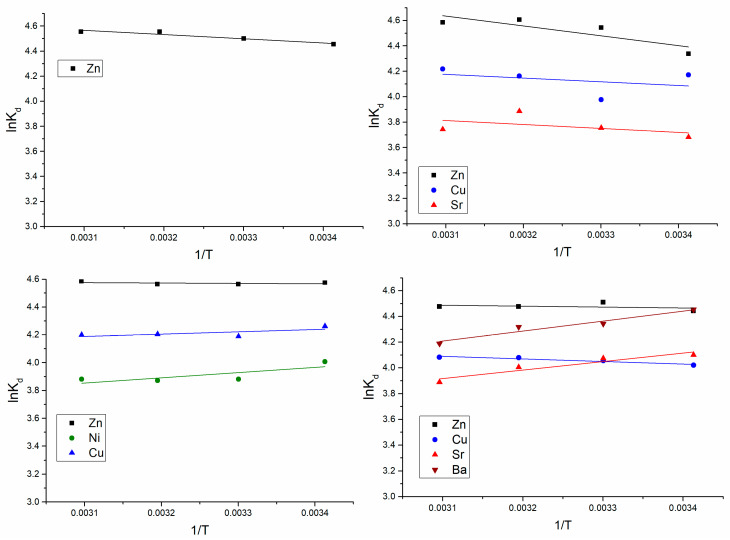
ln*K_d_* versus 1/*T*.

The values of Δ*H*° and Δ*S*° were determined from the linear van’t Hoff plot (Figure 4) of ln*K_d_* versus 1/*T* slope. 

The calculated parameters are given in Table 5.

The negative values of Δ*G*° for all metal ions present in the analyzed systems showed that biosorption process was spontaneous in nature. The enthalpy change, Δ*H*°, for Ni(II) and Cu(II) in the Zn(II)-Cu(II)-Ni(II) system, and Sr(II) and Ba(II)) in Zn(II)-Cu(II)-Sr(II)-Ba(II), was negative, indicating the exothermic reaction. For the rest of the elements, positive Δ*H*° values were obtained, thus confirming the endothermic nature of the process. Positive values of entropy, Δ*S*°, were obtained for all elements in the analyzed systems. 

#### 3.1.5. FTIR Analysis

The interaction of metal ions with yeast biomass was also investigated by the FTIR technique. FTIR spectrum of control biomass *S. cerevisiae* biomass (Supplementary Materials Figure S4) shows absorption bands in the regions 1020 and 1510 cm^−1^. The band at a position of about 1020 cm^−1^ appertains to C–O stretching of alcoholic compounds, polysaccharides (or C–N stretching, C–H deformation), while the band at about 1510 cm^−1^ is attributed to N–O asymmetric stretching, C=C stretching (in-ring), C–H asymmetric deformation, or vibration of the CH_3_ group. The peaks at 1225, 1400, and 2800 cm^−1^ are related to the vibration of carboxyl (C=O) and alkyl (–CH_3_ or CH_2_) groups, while peaks at 1092 and 1043 cm^−1^ correspond to the phosphodiester groups and vibration of polysaccharide skeleton. The peak at 1730 cm^−1^ is related to the vibration of C=C groups of alkenes and C=O from the acetyl groups. The peaks at 3600–3200 cm^−1^ are attributed to hydroxyl (–OH) and amine (–NH) groups and at 2950–2800 cm^−1^ to methyl (–CH) groups. The band at 3230 cm^−1^ is relevant to the standard absorption band of the amido group (HN=O). Bands at the 1650–1200 cm^−1^ region could be characteristic to the amide I–III bands of polypeptide/proteins [10].

### 3.2. Metal Removal from Industrial Effluent

**Figure 5:**
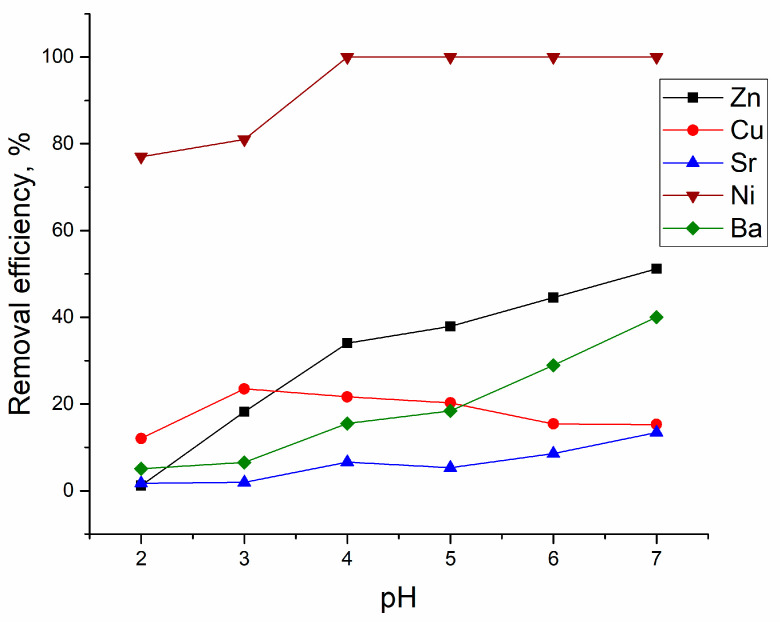
Efficiency of metal ions removal from industrial effluent at different initial pH values (at T 20 °C; sorbent dosage 10 g/L; adsorption time 1 h).

The analyzed effluent contained Zn(II), Sr(II), Cu(II), Sr(II), and Ba(II) ions in different concentrations, and the dominant element was Zn(II). The concentrations of other metal ions in the effluent were significantly lower and were not considered in further discussion. To study the effect of pH on metal removal from the effluent, the pH of the effluent was changed from 2.0 to 6.0. According to data presented in Figure 5, the removal of Zn(II) ions increased over the studied pH, from 1.2% at pH 2.0 to 51% at pH 6.0.

Lower Zn(II) removal in comparison with the batch system can be explained by its higher concentration in the effluent and presence of other metal ions in solution. The same pattern was noticed for Ba(II) and Sr(II) ions, whose removal grew with the increase of pH of the effluent. Yeast removal efficiency toward Ni(II) ions was very high; at pH ≥ 4.0, it was completely removed from the solution. The pH 3.0 was optimal for Cu(II) removal. 

**Figure 6:**
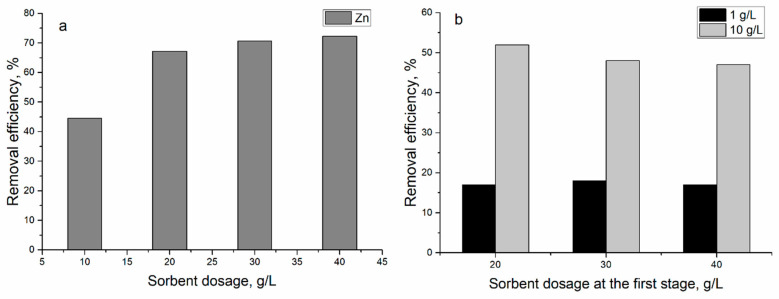
Removal of zinc ions from industrial effluent at different sorbent dosage (at T 20 °C; adsorption time 1 h): (**a**) first stage and (**b**) second stage.

To reduce Zn(II) concentration, the effect of sorbent concentration on Zn(II) removal efficiency was investigated in the subsequent scheme with the addition of new sorbent to the effluent. In the first stage, the concentration of sorbent varied from 10 to 40 g/L. The increase of sorbent concentration led to an increase of yeast removal capacity from 44 to 72% (Figure 6a).

To increase Zn(II) removal to effluent obtained after its interaction with sorbent in concentrations of 20–40 g/L, at the first stage, the new sorbent was added in concentration 1.0 or 10 g/L (Figure 6b). The addition of 1.0 g/L of new biosorbent to the treated effluent resulted in the removal of 17% of Zn(II) ions in all experimental variants, while the addition of 10 g/L of biosorbent led to sorption of 47–52% of Zn(II) onto yeast cells. Therefore, during two cycles, it was possible to remove 72–85% of Zn(II) ions from the effluent.

## 4. Discussion

The effect of different parameters on metal removal from the batch system was investigated. The pH of the solution has a significant impact on metal removal by biological sorbents. The maximum sorption of metal ions present in the studied systems occurred at a pH range of 3.0–6.0. The removal of Zn(II) from the analyzed systems lay in the range of 85–100%, and the highest efficiency of adsorption was achieved in the Zn(II) system. The addition of other metal ions in the analyzed systems resulted in the slight decline of Zn(II) removal by *S. cerevisiae* biomass. In Reference [6], it was shown that, during the simultaneous presence in solution of Ni(II), Cu(II), and Zn(II), *S. cerevisiae* showed higher affinity for Cu(II) ions, while Zn(II) and Ni(II) removal in the ternary system was reduced in comparison with single-element solutions. 

The different pH binding profiles for the heavy metal ions present in the analyzed system can be due to the participation of different groups in metal capture, as well as different concentrations of metal ions present in the analyzed systems. For example, high sorption of Cu(II) at pH 3.0 can be connected with the participation of amino groups, besides carboxyl and hydroxyl groups in Cu(II) capture [13]. An important role in copper binding belongs to carboxylic and amine groups [14]. Carboxyl, amino, hydroxyl, and amide groups participated in Zn, Ni, and Cu sorption by yeast biomass [4]. The mannoproteins and glucans present on the cell wall might be responsible for the biosorption of Ni(II) ions by yeast biomass [15]. Ba(II) can be removed from the solution through its binding to phosphate groups [16]. Strontium biosorption by the yeast biomass occurred mainly due to its exchange with magnesium ions, which content in biomass can be reduced up to 40-fold [17]. Zinc biosorption by *S. cerevisiae* led to a decrease of potassium and magnesium content in biomass [18]. Calcium and sodium were released in a considerable amount at copper biosorption by *Sargassum filipendula* [19]. 

Differences in metal uptake can also be associated with the metal chemistry and the properties of biosorbent. In Reference [7], it was shown that metal biosorption increased with the rise of valence and atomic number of metals as follows: ^82^Pb > ^28^Ni > ^24^Cr.

At pH 2, for all analyzed systems, metals biosorption was insignificant; this can be explained by a high concentration of protons, which compete with metal cations for binding sites [1,5]. With the pH increase, the cell surface becomes more negatively charged, resulting in the increase of the affinity to metal ions in cationic form. At pH 6.0, *Candida rugosa* biofilm showed maximum Zn(II) removal of 44%, whereas *Cryptococcus laurentii* biofilm removed 37% of Zn(II) [3]. Cu^2+^ and Zn^2+^ were effectively removed from aqueous solutions by *S. cerevisiae* crosslinked with formaldehyde at pH 5.3 and 6.0, respectively [8]. Optimal pH for Pb, Zn, Cr(III), Cr(VI), Cd, and Cu uptake by yeast was greater than pH 5.0 [9]. 

Kinetic studies are important in biosorption experiments since they allow us to determine the contact time required for maximum removal of metals from the effluents [4]. The biosorption of metal ions present in the analyzed system was very quick. The optimal time to achieve maximum metal removal varied between 30 min and 1 h, and then the equilibrium was achieved for the main part of elements, except Cu(II) in the Zn(II)-Ni(II)-Cu(II) system, and this can be explained by its desorption and the appearance of new binding sites for Zn(II) and Ni(II) [20]. Biosorption of Pb, Zn, Cr, Co, Cd, and Cu onto yeast *S. cerevisiae* increased sharply in 30 min and then reached the equilibrium [1]. The sorption of Pb^2+^, Ag^+^, Cu^2+^, Zn^2+^, Co^2+^, and Sr^2+^ by yeast took place in 10 min of contact, and it took 1 h for Cs^+^. The removal efficiencies of *S. cerevisiae* biomass toward Pb^2+^, Ag^+^, Cu^2+^, Zn^2+^, Co^2+^, Sr^2+^, and Cs^+^ was very different and varied from 9 to 80% [21]. 

Three kinetic models were applied to describe experimentally obtained data. The Elovich model is applied to describe adsorption on heterogeneous surfaces and also indicates that chemisorption is one of the dominant mechanisms of metal sorption [22]. The model was applicable to describe the data obtained for Zn(II) and Cu(II) in the Zn(II)-Cu(II)-Sr(II)-Ba(II) system. The pseudo-second-order kinetic model indicates that metal removal occurs thought chemisorption [22]. The model was applicable to describe experimentally obtained data for Zn(II) in three systems from four analyzed, as well as for other elements (Table 3). The pseudo-first-order model suggests one-site-occupancy adsorption [23]. For elements present in analyzed systems, except for Cu(II) in Zn(II)-Cu(II)-Sr(II), the coefficient of determination obtained for pseudo-first-order and pseudo-second-order models was higher than 0.94, indicating the applicability of both models for the description of experimentally obtained data. It should be mentioned that values of *SSE* (Table 3) calculated for pseudo-second-order and pseudo-second order models were similar, indicating good fitness of the data by proposed models. Another parameter which allows us to determine the applicability of kinetic model is adsorption rate value. At almost similar R^2^ and *SSE*, the adsorption rate values for pseudo-first order model were lower than for the pseudo-second-order model, indicating a lower rate of adsorption [24]. 

The adsorption rate values are dependent on initial sorbent and metal ions concentrations. Thus, Solisio et al. (2008) [25] showed that, with the increase of the adsorbent concentration from 1.0 to 4.0 g/L, the cadmium adsorption rate grew, while the increase of cadmium concentration by more than 400 mg/L resulted in the decrease of adsorption rate. Martins et al. (2014) [26] studying cadmium and lead removal by *Fontinalis antipyretica* showed that the adsorption rate of the pseudo-first-order model significantly decreased as the initial cadmium and lead concentration increased up to 100 mg/L, while for the pseudo-first-order model, it increased for cadmium and decreased for the lead with the rise of metal ions concentration. 

Metal binding to highly heterogeneous systems can occur concurrently by several mechanism [22]. Since the pseudo-second-order and Elovich models were applicable for the description of experimentally obtained data, metal adsorption by biomass can occur in two steps: (i) metal ions transfer to the binding sites and (ii) interaction with the binding sites (chemical complexation or ion exchange) [22]. Heavy metals bind to yeast biomass can occupy lectin Ca^2+^-binding sites on the cell surface [4]. In different research on metal biosorption, using yeast mainly, applicability of pseudo-second-order equation for description of experimentally obtained data was shown. The pseudo-second-order equation better described the kinetics of Pb^2+^, Ag^+^, Cu^2+^, Zn^2+^, Co^2+^, Sr^2+^, and Cs^+^ sorption onto waste *S. cerevisiae* biomass [21]. The pseudo-second-order model provides the best correlation of the data for Pb, Zn, Cr, Co, Cd, and Cu biosorption onto yeast *S. cerevisiae* [1]. 

The equilibrium of sorption is of great importance for understanding the sorption process [14]. The increase of Zn(II) ions concentration in solution from 10 to 100 mg/L resulted in an increase of its sorption by biomass. The maximum sorption capacity of *S. cerevisiae* for Zn(II) ions was almost the same in all analyzed systems, while the sorption of other metal ions did not depend on Zn(II) concentration in solution. The metal uptake capacities of the *Saccharomyces cerevisiae* crosslinked with formaldehyde for Cu^2+^, Zn^2+^, and Cd^2+^ were found to be 8.0, 7.1, and 14.0 mg/g, respectively [8]. The high values of determination coefficients obtained for the Langmuir isotherm model suggest homogeneous adsorption within the adsorbent and formation of the monolayer [1,27]. In Zn(II)-Cu(II)-Sr(II), and Zn(II)-Cu(II)-Sr(II)-Ba(II) systems, the Freundlich isotherm model was also suitable to describe experimentally obtained data. The Freundlich isotherm model is applicable for description of adsorption on heterogeneous surfaces and suggests that sorption energy exponentially decline upon the saturation of the sorption centers [27]. Since coefficients of determination for both models were high, it can be suggested that Zn(II) biosorption by *S. cerevisiae* follows an intermediate behavior between mono- and a multilayer adsorption mechanism [28].

In all analyzed systems, the *q_e_* was found to be lower than *q_m_*, indicating that the surface of the microorganism during Zn(II) biosorption was not fully covered [7]. The Langmuir model was found to be more appropriate to describe Pb^2+^, Ag^+^, Cu^2+^, Zn^2+^, Co^2+^, Sr^2+^, and Cs^+^ biosorption by yeast biomass [21]. Both the Langmuir and Freundlich models fit well for adsorption data for Pb, Zn, Cr, Co, Cd, and Cu ions [1].

The increase of the temperature from 20 to −50 °C did not significantly affect metal adsorption in analyzed systems, as its increase or decrease was not more than 20%. Ozer and Ozer [7] studying the effect of temperature on Pb(II), Ni(II), and Cr(VI) ions’ uptake by *S. cerevisiae* showed that an increase of *S. cerevisiae* uptake capacity at the temperature range of 15–25 °C and its decrease with rise of the temperature up to 40 °C, probably due to the damage of active binding sites in the biomass. According to the data presented in Table 5, it is seen that the biosorption process in analyzed systems was spontaneous. The values of energy Δ*G*° between 0 and 20 kJ/mol point out that the adsorption process is physical sorption [29]. Positive ∆Hº values obtained for Zn(II) in all analyzed systems, as well as the Cu(II) and Sr(II) in Zn(II)-Ni(II)-Cu(II) system and Cu(II) in Zn(II)-Cu(II)-Sr(II)-Ba(II), indicate the endothermic character of metal biosorption, while for rest of metal ions, it was exothermic. Since ∆*H*° was less than 25 kJ/mol, sorption can be considered as physical [30]. Positive Δ*S*° values obtained for all elements in analyzed systems indicate the increased randomness at the solid/liquid interface during the sorption of metal ions onto the yeast cells, as well as its affinity to analyzed metal ions [21]. In the metal-loaded biomass, a shift by 2–10 cm^-1^ of bands corresponding to OH, C=C, C=O, C–H, C–N, and NH groups indicates their involvement in metal ions’ binding. According to data obtained by NAA, sodium content in biomass loaded with metal ions decreased 1.9–5.5 times in comparison with the control, of potassium 1.3–2.9 times, and of calcium 1.1–1.7 times, thus indicating their participation in the ion-exchange process.

The pH and biomass dosages, as well as their combination, are dominant factors affecting the removal of metal ions from industrial effluents [31]. As in the case of batch systems, the removal efficiency of yeast biomass toward Zn(II) and other metal ions present in the systems increases with the increase of the pH of the solution. Lower Zn(II) removal, in comparison with the batch system, can be explained by its higher concentration in the effluent and presence of other metal ions in the solution. At pH >4.0, Ni(II) was completely removed from the solution. High Ni(II) removal can be explained by its lower concentration (0.8 mg/L), in comparison with the batch system (2.0 mg/L), and competition for binding sites manly with Zn(II) as the concentration of other elements present in the effluent were significantly lower than that of Ni(II) and Zn(II). Variation of the pH values at sorbent concentrations of 10 g/L allowed the reduction of concentrations of all elements, except Zn(II), in the effluent, under the values of maximum permissible concentration (MPC) [32]. It was shown in our previous studies that an increase of sorbent dosage to a certain value increases metal ions’ removal, and then the efficiency of metal removal increases just insignificantly or can even be reduced [33,34]. The applied the subsequent scheme, with the addition of new sorbent to the effluent, resulted in a decrease of Zn(II) concentration in the effluent to 1.5MPC [32]. The optimal scheme to achieve maximal Zn(II) removal is as follows: 20 g/L of yeast biomass on the first stage and 10 g/L on the second stage. Increase of sorbent concentration on the first stage up to 40 g/L had not resulted in a significant increase of Zn(II) removal; thus, is not economically profitable. Metal removal in the subsequent system by yeast biomass was investigated by Hernández Mata et al. [35]. After 40 days of the experiment, a reduction of 81.5% zinc, 76.5% copper, manganese 95.5%, and 99.8% of iron was attained. Sorbent obtained after the biosorption process can be regenerated by using different eluents. However, in the case of treatment of multi-metal, it is a difficult task, since metal ions can be present in the effluent in anionic and cationic forms that require sorbent regeneration in several steps, increasing the price of the process. Another drawback of biological sorbents regeneration is a progressive decrease in the efficiency of metal removal with an increase in the number of cycles due to the structural damage of biosorbent and blockage of binding sites [33]. Thus, yeast being a waste of the beer production process after biosorption experiments can be used as an additive in the production of materials for road construction.

## 5. Conclusions

Biomass of yeast *S. cerevisiae* was used for metal removal from four synthetic and one real zinc-containing effluents. The optimal pH for metal removal lay in the range 3.0–6.0 and allowed removal of 45–100% of metal from the solution, depending on the analyzed system and metal type. The increase of Zn(II) concentration in solution did not affect the sorption of other metal ions present in analyzed systems. Langmuir and Freundlich isotherm models was suitable to describe Zn(II) biosorption with *q_m_* values in the range of 8.99–17 mg/g. By evaluating the coefficients of determination of every kinetic model, it is seen that the adsorption of metals ions onto yeast biomass can be described by the pseudo-first, pseudo-second-order, and Elovich models. Surface adsorption, chemisorption, and ion exchange can be considered as the main mechanisms of metal removal from solution. OH, C=C, C=O, C–H, C–N, and NH groups are involved in the biosorption of metal ions. Varying pH at sorbent concentration of 10 g/L it was possible to reduce the concentration of Cu(II), Ba(II), Sr(II), and Ni(II) below maximum admissible level. The highest efficiency of removal of Zn(II) ions from complex real effluent was achieved in a two-stage subsequent system, with the addition of new biosorbent: 20 g/L of biosorbent at the first stage and 10 g/L of sorbent on the second stage, at pH 6.0 and contact time 60 min. Yeast *S. cerevisiae* showed to be an excellent candidate for the treatment of zinc-containing complex effluents.

## Figures and Tables

**Table 1:** Elemental composition of synthetic effluents.

System	Concentration, mg/L				
	Zn	Cu	Ni	Sr	Ba
Zn(II)	10 ± 0.1	-	-	-	-
Zn(II)-Sr(II)-Cu(II)	10 ± 0.3	5 ± 0.03	-	1 ± 0.01	-
Zn(II)-Ni(II)-Cu(II)	10 ± 0.2	2 ± 0.04	2 ± 0.01	-	-
Zn(II)-Sr(II)-Cu(II)-Ba(II)	10 ± 0.1	2 ± 0.05	2 ± 0.03	-	1 ± 0.03

**Table 2:** Elemental composition of industrial effluent.

Element	Sr	Ni	Cu	Zn	Ba	pH
Concentration, µg/L	340	839	58	49,843	35	6.0

**Table 3:** The constants and determination coefficients (*R*^2^) of the kinetics models.

Systems	Metal	*q_exp_*, mg/g	Model										
			PFO				PSO				EM		
			*q_e_*, mg/g	*k*_1_, min^−1^	*R* ^2^	*SSE*, %	*q_e_*, mg/g	*k*_2_, g/mg·min	*R* ^2^	*SSE*, %	*α*, mg/g·min	*β*, g/min	*R* ^2^
Zn(II)	Zn	1.0	0.96	0.24	0.98	0.05	1.02	0.4	0.99	0.06	3.0	9.9	0.98
Zn(II)-Cu(II)-Sr(II)	Zn	1.0	0.97	0.16	0.99	0.07	1.06	0.22	0.99	0.09	2.6	7.1	0.97
	Cu	0.31	0.03	0.98	0.31	0.007	n.a.	n.a.	n.a.	n.a.	n.a.	n.a.	n.a.
	Sr	0.045	0.046	0.5	0.98	0.001	0.047	4.4	0.99	0.001	n.a.	n.a.	n.a.
Zn(II)-Ni(II)- Cu(II)	Zn	1.2	1.17	0.15	0.97	0.09	1.28	0.18	0.99	0.11	2.7	5.8	0.97
	Ni	0.13	0.14	0.1	0.97	0.87	0.15	1.07	0.97	1.05	1.3	42	0.95
	Cu	0.11	0.14	51	0.77	0.01	n.a.	n.a.	n.a.	n.a.	n.a.	n.a.	n.a.
Zn(II)-Cu(II)-Sr(II)-Ba(II)	Zn	1.1	1.1	0.26	0.97	0.05	1.15	0.4	0.98	0.07	3.2	8.7	0.99
	Cu	0.12	0.11	0.53	0.93	0.004	0.12	4.4	0.96	0.005	8.3	91	0.98
	Sr	0.14	0.13	0.4	0.99	0.003	0.14	7.9	0.99	0.004	n.a.	n.a.	n.a.
	Ba	0.099	0.095	10.3	0.99	9.9 × 10^−4^	0.096	481	0.99	9.7 × 10^−4^	n.a.	n.a.	n.a.

**Table 4:** The parameters for Langmuir, Freundlich, and Temkin adsorption isotherms.

Model	Parameters	System			
		Zn(II)	Zn(II)-Cu(II)-Sr(II)	Zn(II)-Ni(II)-Cu(II)	Zn(II)-Cu(II)-Sr(II)-Ba(II)
Langmuir	*q_m_*, mg/g	8.99	10.4	17	12
	*b*, L/mg	0.002	0.01	0.005	0.009
	*R* ^2^	0.99	0.99	0.99	0.99
Freundlich	*K_F_*, mg/g	0.43	0.25	0.17	0.25
	1/*n*	0.59	0.67	0.77	0.7
	*R* ^2^	0.98	0.99	0.98	0.99
Temkin	*a_T_*, L/g	0.17	0.14	0.11	0.13
	*B*, J/mol	3.26	1.3	1.07	1.1
	*R* ^2^	0.98	0.96	0.92	0.96

**Table 5:** Thermodynamics parameters for metal biosorption on *S. cerevisiae.*

System	Metal	∆*G*°, kJ/mol				∆*H*°, kJ/mol	∆*S*°, J/mol·K
		293 K	303 K	313 K	323 K		
**Zn(II)**	Zn	−10.8	−11.4	−11.8	−12.2	2.9	46.5
**Zn(II)-Cu(II)-Sr(II)**	Zn	−10.6	−11.2	−11.8	−12.4	6.4	58
	Cu	−9.9	−10.4	−10.8	−11.2	2.4	42
	Sr	−9.0	−9.4	−9.8	−10.24	2.6	40
**Zn(II)-Ni(II)-Cu(II)**	Zn	−11.1	−11.5	−11.9	−12.3	0.2	39
	Ni	−9.7	−9.9	−3.9	−10.1	−10.4	22
	Cu	−10.4	−10.7	−11.0	−11.3	−1.4	31
**Zn(II)-Cu(II)-Sr(II)-Ba(II)**	Zn	−10.9	−11.2	−11.6	−12.0	0.6	39
	Sr	−10.1	−10.3	−10.4	−10.6	−5.5	16
	Cu	−9.8	−10.2	−10.4	−10.6	1.7	39
	Ba	−10.8	−11.0	−11.1	−11.3	−6.4	15

## Supplementary Materials

The following are available online at https://www.mdpi.com/1996-1944/13/16/3624/s1, Figure S1: Kinetics of the metal adsorption using *S. cerevisiae* in Zn(II)-Sr(II)-Cu(II) system, Figure S2: Kinetics of the metal adsorption using *S. cerevisiae* in Zn(II)-Ni(II)-Cu(II) system, Figure S3: Kinetics of the metal adsorption using *S. cerevisiae* in Zn(II)-Cu(II)-Sr(II)-Ba(II) system, Figure S4: FTIR spectrum of *S. cerevisiae* biomass before and after metal biosorption.
