# Supplementary material for: Efficient Removal of Metals from Synthetic and Real Galvanic Zinc–Containing Effluents by Brewer’s Yeast Saccharomyces cerevisiae
Source: Materials (Basel). 2020 Aug 16;13(16):3624. doi: 10.3390/ma13163624 (PMC7475842; doi:10.3390/ma13163624)
Supplement: Supplementary file 1 [file materials-13-03624-s001.pdf]

materials

Efficient Removal of Metals from Synthetic and Real Galvanic Zinc–Containing Effluents by Brewer's Yeast *Saccharomyces Cerevisiae*

Supplementary Information

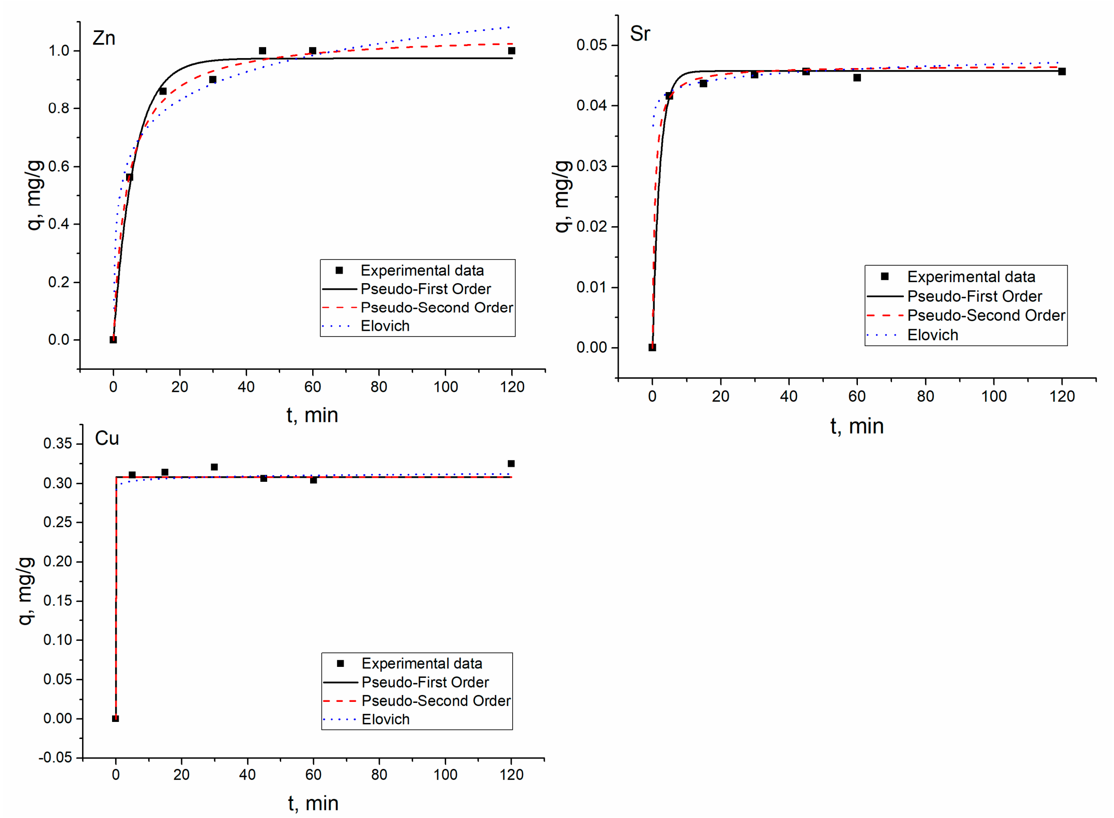

Figure S1. Kinetics of the metal adsorption using *S. cerevisiae* in Zn(II)-Sr(II)-Cu(II) system.

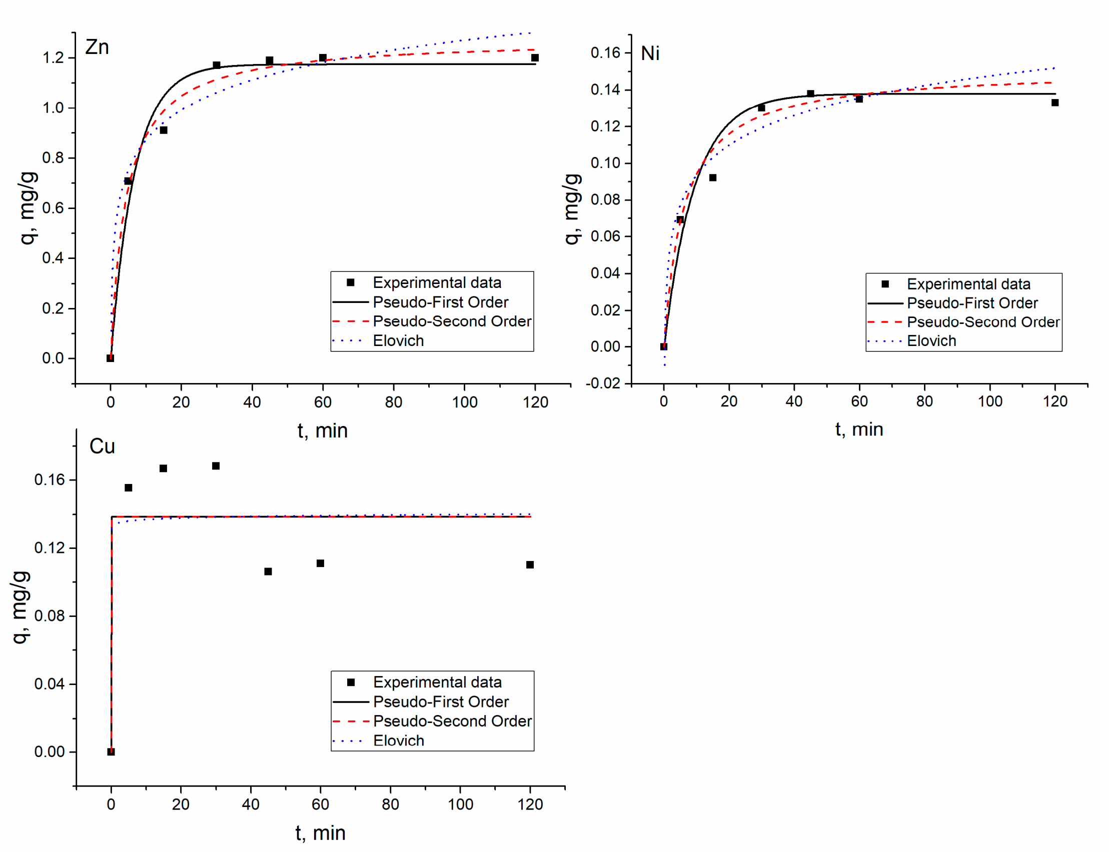

Figure S2. Kinetics of the metal adsorption using *S. cerevisiae* in Zn(II)-Ni(II)-Cu(II) system.

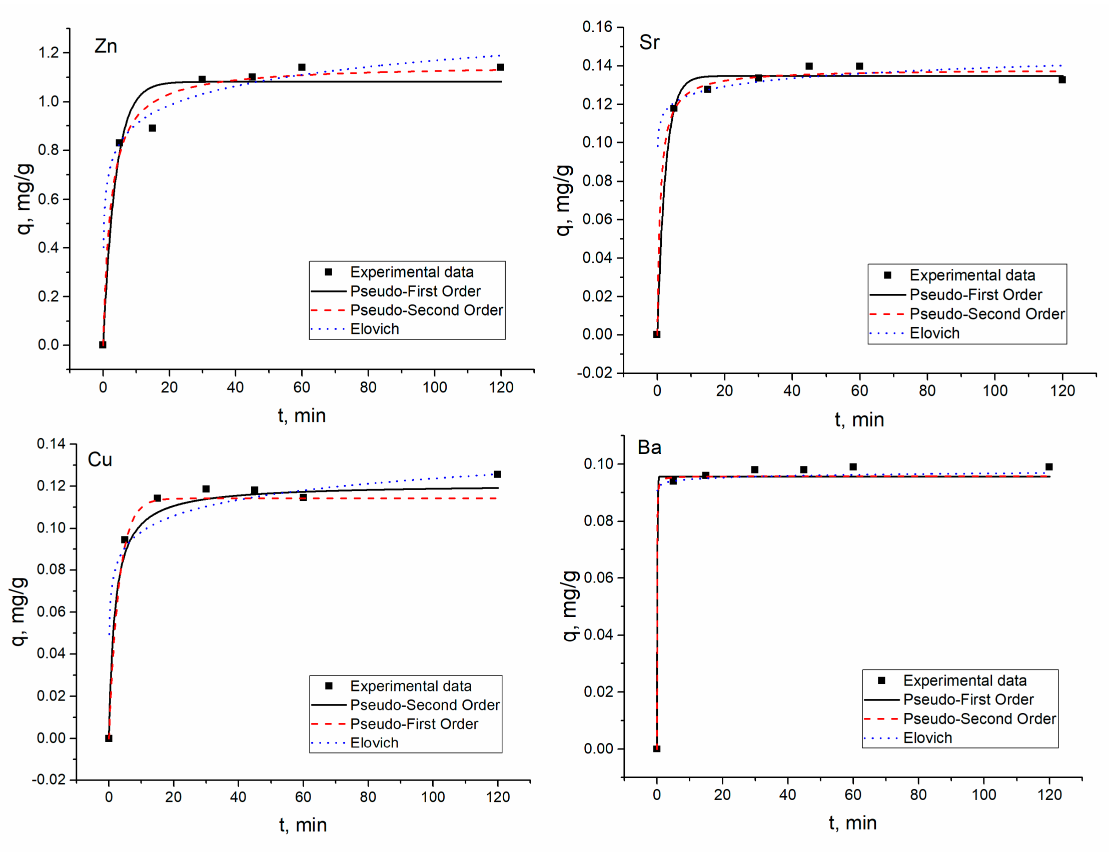

Figure S3. Kinetics of the metal adsorption using *S. cerevisiae* in Zn(II)-Cu(II)-Sr(II)-Ba(II) system.

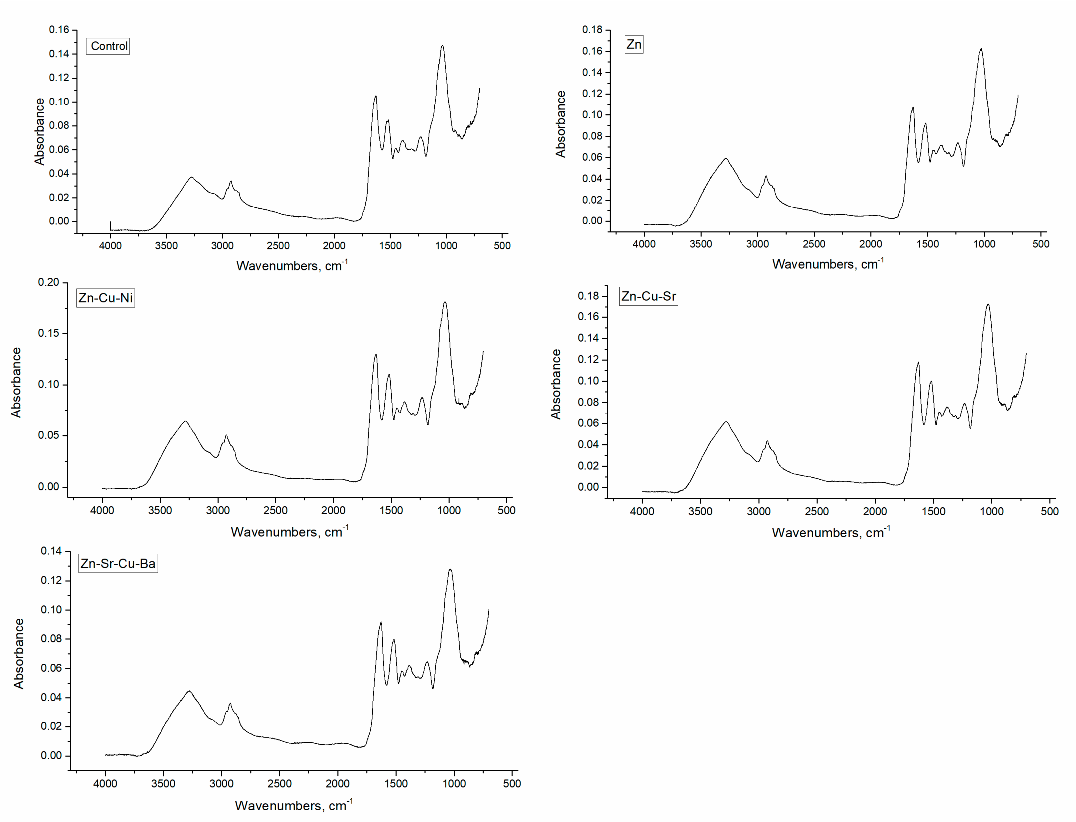

Figure S4. FTIR spectrum of *S. cerevisiae* biomass before and after metal biosorption.
